# Species-Specific miRNAs in Human Brain Development and Disease

**DOI:** 10.3389/fncel.2019.00559

**Published:** 2019-12-18

**Authors:** Kanella Prodromidou, Rebecca Matsas

**Affiliations:** Laboratory of Cellular and Molecular Neurobiology—Stem Cells, Department of Neurobiology, Hellenic Pasteur Institute, Athens, Greece

**Keywords:** RNA sequencing (RNAseq), primate, neurogenesis, evolution, gene networks, brain, miRNAs, human

## Abstract

Identification of the unique features of human brain development and function can be critical towards the elucidation of intricate processes such as higher cognitive functions and human-specific pathologies like neuropsychiatric and behavioral disorders. The developing primate and human central nervous system (CNS) are distinguished by expanded progenitor zones and a protracted time course of neurogenesis, leading to the expansion in brain size, prominent gyral anatomy, distinctive synaptic properties, and complex neural circuits. Comparative genomic studies have revealed that adaptations of brain capacities may be partly explained by human-specific genetic changes that impact the function of proteins associated with neocortical expansion, synaptic function, and language development. However, the formation of complex gene networks may be most relevant for brain evolution. Indeed, recent studies identified distinct human-specific gene expression patterns across developmental time occurring in brain regions linked to cognition. Interestingly, such modules show species-specific divergence and are enriched in genes associated with neuronal development and synapse formation whilst also being implicated in neuropsychiatric diseases. microRNAs represent a powerful component of gene-regulatory networks by promoting spatiotemporal post-transcriptional control of gene expression in the human and primate brain. It has also been suggested that the divergence in miRNA expression plays an important role in shaping gene expression divergence among species. Primate-specific and human-specific miRNAs are principally involved in progenitor proliferation and neurogenic processes but also associate with human cognition, and neurological disorders. Human embryonic or induced pluripotent stem cells and brain organoids, permitting experimental access to neural cells and differentiation stages that are otherwise difficult or impossible to reach in humans, are an essential means for studying species-specific brain miRNAs. Single-cell sequencing approaches can further decode refined miRNA-mRNA interactions during developmental transitions. Elucidating species-specific miRNA regulation will shed new light into the mechanisms that control spatiotemporal events during human brain development and disease, an important step towards fostering novel, holistic and effective therapeutic approaches for neural disorders. In this review, we discuss species-specific regulation of miRNA function, its contribution to the evolving features of the human brain and in neurological disease, with respect also to future therapeutic approaches.

## Introduction

Human brain development presents unique features and underlies the intricately coordinated spatiotemporal expression of thousands of genes. This elaborate mechanism entails the timely acquisition of diverse cellular identities further orchestrating regional specialization and inter-connectivity in the brain. microRNAs (miRNAs) are powerful post-transcriptional regulators, increasingly recognized as important components of fundamental neurodevelopmental processes and related disorders (Adlakha and Saini, 2014). miRNAs act to repress the translation or degrade their mRNA targets to modulate and fine-tune gene expression levels. One of the early roles ascribed to miRNAs was their contribution to developmental transitions by suppressing transcripts associated with the previous stage. An alternative, but complementary hypothesis suggests that miRNAs act reduce the variance in the expression level of their target genes, conferring increased robustness to signaling decisions during development (Hornstein and Shomron, 2006; Ebert and Sharp, 2012). Importantly, it is appreciated that miRNA-driven regulation contributed critically to gene expression changes on the human evolutionary lineage affecting genes involved in progenitor proliferation and neuronal generation and function (Nowakowski et al., 2013; Arcila et al., 2014). miRNA-mediated regulation in the developing brain presents primate distinct aspects, including over 100 primate-specific and 14 human-specific miRNAs that have been identified (Berezikov, 2011; Hu et al., 2011).

In this review we present current data on species-specific miRNA regulation and discuss miRNAs as hubs of critical brain transcriptional processes during human neural development. Finally, we stress the requirement to delineate the functional significance of miRNA-driven transcriptome changes at the single-cell level, as an important step towards resolving the complex regulatory network operating during human neurogenesis. Towards this direction, brain organoids and application of single-cell sequencing methodologies constitute invaluable tools to address causality between the emergence of novel miRNAs and rewiring of transcriptional programs during the evolution of brain complexity.

## Distinct Features of Human Brain Development

**Figure 1:**
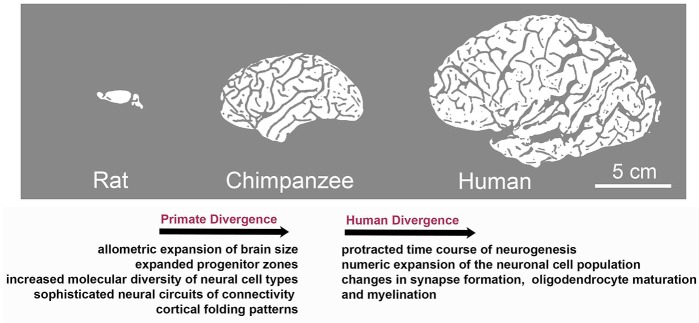
Species-specific evolution of traits associated with brain development during the divergence to primate and human lineages. Although brain development follows the same basic principles across mammals, evolution has resulted in the appearance of species-specific features. Characteristics of primate divergence include allometric increase in brain size, expansion of progenitor zones along with increased diversity of neural cell types and sophistication of neural circuits reflected in enhanced gyral anatomy. Human brain development is further distinguished by a relatively protracted period of neurogenesis, followed by an extraordinary numeric expansion of the neuronal cell population and by the heterochronic or heterotopic expression of genes associated with synapse formation and myelination in brain regions including the prefrontal cortex, which is central to human cognition and behavior.

Although brain development follows the same principles across mammals, the primate and human central nervous system (CNS) is distinguished by highly derived features. These include expanded progenitor zones (Smart et al., 2002) accompanied by enhanced and tightly controlled proliferative and/or neurogenic potential of progenitor cells (Otani et al., 2016; Sousa et al., 2017a,b), further associated with molecular changes and an increased diversity of neural cell types (Bystron et al., 2006; Lui et al., 2011; Gulden and Šestan, 2014; Taverna et al., 2014; Bae et al., 2015; Dehay et al., 2015). Human brain development is characterized by a relatively protracted time course of neurogenesis, followed by an extraordinary numeric expansion of the neuronal cell population (Rockel et al., 1980; Hutsler et al., 2005; Marín-Padilla, 2014; Otani et al., 2016) and the emergence of sophisticated neural circuits of connectivity reflected in prominent changes in gyral anatomy (Rogers et al., 2010; Hofman, 2012; Figure 1).

Brain evolution produced changes in morphology, abundance, and function of cell types. For example, differences between rodent and human neuronal features include distinctive membrane (Wang et al., 2015; Eyal et al., 2016) and synaptic properties Molnár et al., 2008, 2016; Testa-Silva et al., 2010; Verhoog et al., 2013; Szegedi et al., 2016). Deviations between humans and non-human primates (NHPs) have also been reported regarding the morphology and number of glial cells in the brain (Oberheim et al., 2012; Bianchi et al., 2013; Herculano-Houzel et al., 2016). In addition, excitatory projection neurons in humans show more elaborate dendritic arborization, and contain a greater number and density of spines compared to non-human primates (NHPs; Duan et al., 2003; Elston et al., 2011). Along this line, a subgroup of modified pyramidal neurons, known as spindle or von Economo neurons, mainly found in the fronto-insular and anterior cingulate cortex, are larger and more numerous in humans than in other apes (Allman et al., 2010). Although their function remains elusive, they have been implicated in brain disorders with social-emotional deficits, such as autism spectrum disorders (ASDs), schizophrenia (SCZ), frontotemporal dementia and Alzheimer’s disease (AD; Yang et al., 2019). Evidence also exists for species-specific differences in serotonergic transmission, dopamine innervation and the regional localization and abundance of certain subclasses of inhibitory neurons and their axonal projections in the neocortex of humans and NHPs (Sherwood et al., 2004; Raghanti et al., 2008, 2016). Similarly, a specialized GABAergic neuron subtype has recently been identified with a molecular and anatomical signature specific to humans (Boldog et al., 2018).

## Gene Networks Associated With Primate- and Human-Specific Brain Adaptations

Comparative genomic studies revealed that adapted brain specializations and capacities may be partly explained by human-specific gene conversions that impact the function of proteins associated with neocortical expansion, synaptic function, and language development. Characteristic examples include genes that have undergone human-specific duplication like the cortical development gene Slit-Robo Rho GTPase activating protein 2 (*SRGAP2*) which induces branching of neurons and neurite outgrowth (Dennis et al., 2012). Another case is ARHGAP11 which is expressed in basal progenitors and promotes neocortical expansion by increasing neuron numbers and brain folding (Florio et al., 2015). *FOXP2* is also a gene that has undergone surprisingly rapid evolution in the primate lineage leading to humans and encodes a homeodomain protein essential for normal human speech (Lai et al., 2001). Similarly, *ASPM* (Gai et al., 2016), *Microcephalin* (Evans et al., 2004) and *AHI1* genes (Ferland et al., 2004) that have undergone “positive selection,” encode for human proteins that associate with normal cerebral cortical size and axon guidance.

Importantly, gene co-expression analyses have revealed that transcriptional regulation and complexity in the neocortex have dramatically increased on the human lineage (Konopka et al., 2012; Silbereis et al., 2016). The allometric expansion of the brain is primarily accompanied by changes in patterns of gene networks and neuronal activity leading to the structural reorganization of the connectome and possibly harboring new behavioral and cognitive phenotypes (Buckner and Krienen, 2013). In this regard, recent transcriptome studies have provided crucial information on divergent patterns of molecular expression that are most relevant in evolutionary terms and can be used to uncover human specializations of brain structure and function.

Specifically, fetal gene co-expression modules have been identified that showed substantial regional differences in the developing human neocortex (Johnson et al., 2009; Miller et al., 2014; Pletikos et al., 2014). Enriched genes were shown to be critical for neuronal processes, such as differentiation, maturation, axonal projection, and synapse formation. These molecular networks also displayed divergence between humans and rhesus macaques (Figure 1; Pletikos et al., 2014), highlighting evolving biological processes involved in the patterning and differentiation of neural circuits in discrete areas (Miller et al., 2014; Hoerder-Suabedissen and Molnár, 2015).

Further work revealed human-distinct temporal progression of neurodevelopmental processes predominantly during the early and mid-fetal period. The identified gene modules were linked to synapse formation, neuronal differentiation, oligodendrocyte maturation, and myelination, and exhibited heterochronic or heterotopic expression in brain regions including the prefrontal cortex, which is central to human cognition and behavior (Figure 1). Interestingly, divergent spatiotemporal expression patterns included also genes associated with ASDs and schizophrenia (Konopka et al., 2012; Zhu et al., 2018). These studies highlight gene expression regulation in space and time as an evolution-modified feature that impacts on neurodevelopmental processes as well as in brain complexity and disease. Diverse expression patterns responsible for interspecies differences can be attributed to changes in DNA methylation, histone modifications (Maze et al., 2014), alternative splicing events, promoter-driven transcription regulation (Davuluri et al., 2008; Nilsen and Graveley, 2010) and non-coding RNAs.

## microRNAs Are Powerful Post-Transcriptional Regulators of Gene Expression in the Brain

microRNAs (miRNAs) are a class of short non-coding RNAs of ~22 nucleotides in length that constitute an important component of the regulatory circuitry determining expression patterns. Mature miRNAs mediate post-transcriptional regulation of gene expression through direct degradation of their target mRNA and/or suppression of translation. miRNAs bind to their mRNA targets by partial complementarity between the mRNA’s 3′UTR and a 6–8 nucleotides long “seed” sequence at the 5′ end of the microRNA. Therefore, a single microRNA can target multiple mRNAs simultaneously, while a single mRNA may be regulated by different microRNAs (Klein et al., 2005; Kosik, 2006; Saliminejad et al., 2019).

miRNA biogenesis begins with the transcription of double-stranded primary miRNA (pri-miRNA) short hairpin structures by RNA polymerase II. The pri-miRNA is then cleaved by the RNase-III enzyme, Drosha, producing ~70-bp pre-miRNAs that are exported from the nucleus into the cytoplasm by Exportin 5 (Exp5), a Ran-GTP dependent Nucleo/cytoplasmic cargo transporter. The Dicer enzyme cleaves pre-miRNA sequences into 21–23 nt mature miRNA double-stranded duplexes which are loaded into a pre-RISC (pre-miRNA-induced silencing complex) containing Argonaute (Ago) and other proteins. In the mature miRISC complex the “passenger” strand (complementary strand) is removed leaving just the “guide” strand (mature miRNA strand) which will bind to the mRNA target and instigate inhibition of its expression, reviewed in Winter et al. (2009) and Davis et al. (2015).

The concentration of miRNAs within cells is regulated at different levels. It has been shown that Ago proteins are not only critical for miRNA biogenesis and function, but they also regulate the abundance of mature miRNAs by increasing their stability (Grishok et al., 2001; Diederichs and Haber, 2007; Winter and Diederichs, 2011). Association with mRNA targets also enhances miRNA stability, a phenomenon known as target-mediated miRNA protection, while the introduction of additional target sites can also promote miRNA accumulation (Chatterjee et al., 2011). On the other hand, miRNAs are subject to degradation. It has been shown that miRNA-mRNA interactions not only stabilize but can also destabilize the miRNA and promote its degradation through a process known as target RNA directed miRNA degradation (Ameres et al., 2010; Fuchs Wightman et al., 2018). The degree of sequence complementarity infers the outcome of the miRNA-mRNA interaction, with higher complementarity favoring miRNA degradation and lower complementarity favoring miRNA stabilization. As demonstrated, target mRNAs promote posttranscriptional modifications to the 3′ end of the miRNA involving either the addition of non-templated nucleotides, a process known as “3′-end tailing” or elimination of nucleotides *via* 3′-to-5′ trimming, both of which control the rate of miRNA decay (Baccarini et al., 2011; Marcinowski et al., 2012).

The ability of miRNAs to fine-tune gene expression levels (Schratt, 2009) constitutes a critical property for controlling spatiotemporal events during brain development. Of the 2,500 mature miRNAs that have been identified in humans (Friedländer et al., 2014), an estimated 70% is expressed in the nervous system (Adlakha and Saini, 2014). miRNAs have emerged as important post-transcriptional regulators of gene expression involved in neurogenesis and neural function in mammalian species (Davis et al., 2015; Nowakowski et al., 2018). A relatively small number of brain miRNAs are well characterized, including miR-92 which targets EOMES (TBR2), a T-box transcription factor that is preferentially expressed in cortical intermediate progenitors and regulates cortical neuron production and expansion, thereby affecting the thickness of the cerebral cortex (Nowakowski et al., 2013). miR-124 and miR-9 have also been shown to affect neural lineage differentiation by downregulating multiple mRNAs (Krichevsky et al., 2006). MiR-9 is a highly brain enriched miRNA that is involved in a negative feedback loop with TLX, a nuclear receptor (Zhao et al., 2009) which controls stem cell proliferation in developing and adult brain (Shi et al., 2004; Liu et al., 2008; Zhang et al., 2008). MiR-9 together with miR-124, one of the most abundant miRNAs in the brain, target REST which opposes neuronal differentiation (Conaco et al., 2006; Visvanathan et al., 2007), while REST itself acts as an inhibitor of miR-124 expression. Moreover, both miRNAs act synergistically to repress BAF53a, a subunit of the neural-progenitor-specific BAF (npBAF) chromatin-remodeling complex, operating during the post-mitotic phase of neuronal development (Yoo et al., 2009). Finally, miR-124 downregulates the RNA-binding protein Ptbp1, a repressor of neuron-specific splicing (Makeyev et al., 2007).

Additionally, miRNAs have been shown to be essential for neural subtype specification. For example, miR-7a promotes oligodendrocyte generation by targeting Pax6 and NeuroD4 (Zhao et al., 2012), while miR-218 is required to establish motor neuron fate (Thiebes et al., 2015). miRNAs have also important roles in synapse formation and plasticity. These include miR-125 which targets the post-synaptic protein PSD-95 in cortical neurons (Muddashetty et al., 2011), the neuron-specific miR-129 which represses Kv1.1, a voltage-gated potassium channel that regulates excitability (Sosanya et al., 2013), and miR-219 which downregulates CamKII, a the major mediator of Long Term Potentiation (LTP) and *N*-methyl-D-aspartate receptor (NMDA) signaling (Kocerha et al., 2009). Other miRNAs modulate synaptic function upon activation. miR-485 expression is increased following neuronal stimulation to regulate the pre-synaptic protein SV2A and inhibit neurotransmitter release (Cohen et al., 2011). miR-132, on the other hand, accumulates in response to activity in forebrain neurons to regulate dendritic growth, activity-induced spine growth and spine morphology (Magill et al., 2010; Nudelman et al., 2010).

## The Evolving Role of Mirnas in the Primate and Human Brain

### The Evolution of miRNAs

Evolution of miRNAs is an ongoing process and experimental evidence suggests that along with highly conserved miRNAs, a number of new brain miRNAs have emerged. Many of these are not conserved beyond primates, indicating their recent origin (Berezikov et al., 2006). Following evolutionary adaptations, more than 100 primate-specific miRNAs (that is, miRNAs present only in humans and non-human primates) and 14 human-specific miRNAs (Table 1) have been identified in the developing brain (Berezikov, 2011). Novel miRNAs arise either by the appearance of transcribed hairpin structures or by mutations in the miRNA seed region (Lu et al., 2008). The genomic sources for the acquisition of novel miRNAs are reviewed in detail in “Evolution of microRNA diversity and regulation in animals” (Berezikov, 2011). Briefly, novel miRNAs can emerge by duplication of existing miRNA genes. Alternatively, introns are a frequent source of unstructured transcripts that can gradually evolve into novel intronic miRNAs. *De novo* emergence of miRNAs can also occur, where an evolved transcriptional unit provides a source of the initially unstructured transcript that transitions through the miRNA-like hairpin stage and evolves into a novel miRNA gene. In addition, transposable elements or structured transcripts, such as tRNA and small nucleolar RNA (snoRNA), can provide novel transcriptional units for the evolution of miRNA-like hairpins into novel miRNA genes. Finally, antisense transcription of existing miRNA loci can lead to the formation of miRNA hairpins with novel mature miRNA sequences.

**Table 1:** List of human-specific miRNAs identified so far according to the study of Hu et al. (2012).

Precursor id	Chromosome
hsa-mir-1302-10	chr15
hsa-mir-1302-11	chr19
hsa-mir-1302-2	chr1
hsa-mir-3156-3	chr21
hsa-mir-3648	chr21
hsa-mir-3673	chr8
hsa-mir-3690	chrX
hsa-mir-4487	chr11
hsa-mir-4739	chr17
hsa-mir-5095	chr1
hsa-mir-659	chr22
hsa-mir-941-1	chr20
hsa-mir-941-3	chr20
hsa-mir-941-4	chr20

### miRNAs as Key Regulators in Shaping Patterns of Gene Expression in the Developing and Adult Primate and Human Brain

A series of observations support the notion that miRNAs are instrumental contributors to the alterations that may account for the accelerated evolution of the human brain. First, elevated production of mature miRNAs has been observed in the human brain compared to other species, which is attributed to the higher processing efficiency of miRNA precursors in humans (Chakraborty et al., 2018).

**Figure 2:**
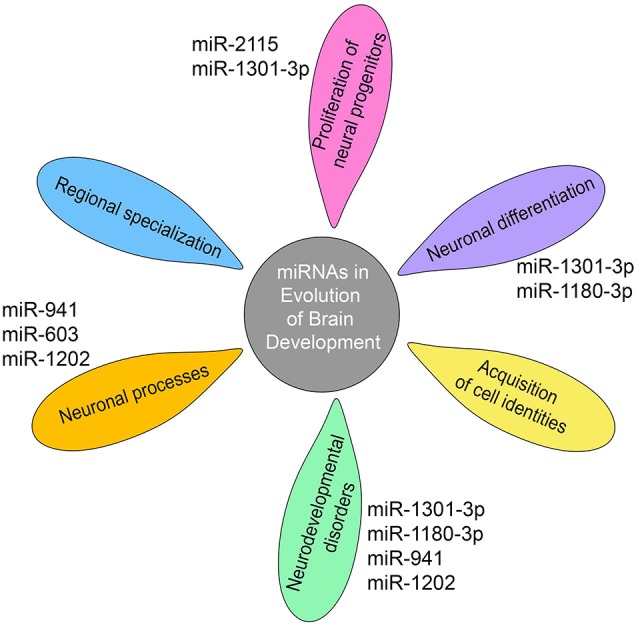
miRNAs shape gene networks during the evolution of human and non-human primate brain development. Transcriptomic studies show that miRNA-mediated regulation during primate brain evolution contributed critically in shaping gene networks associated with progenitor proliferation, neuronal differentiation, and acquisition of cell identities, an extension of neuronal processes, regional specialization and neurodevelopmental disorders as depicted on the petals. A limited number of primate- or human-specific miRNAs are indicated next to the petals, for which separate studies exist so far to demonstrate their individual involvement in the respective biological processes.

It has been proposed that the evolution of miRNA-mediated regulatory networks has contributed to organismal complexity (Berezikov, 2011). This can mechanistically be explained by the ability of miRNAs to advance network functionalities by delivering the extra precision required to constrain the intrinsically noisy gene expression process (Raj and van Oudenaarden, 2008; Herranz and Cohen, 2010). In support, the work of Arcila et al. (2014) on primate-specific miRNAs suggests that integration of novel miRNAs into ancient gene circuitry exerted additional regulation over-proliferation of neural progenitors in cortical germinal areas (Figure 2), a region that demonstrates significant expansion across brain evolution (Arcila et al., 2014).

Furthermore, evolving miRNA-mediated regulation shows critical implications in shaping gene networks and even determining anatomical regions during brain development. Work on primates has revealed that miRNA profiles can resolve discrete areas within the developing cortex while dominant differences were observed between the germinal zone and the differentiated cells in the cortical plate (Arcila et al., 2014). Consistently, studies on the transition from infant to adolescent human brain show differential expression of miRNAs within and between brain regions, with the prefrontal cortex, the region mostly connected with human cognition, exhibiting the greatest number of differentially expressed miRNAs (Ziats and Rennert, 2014). Moreover, miRNA expression displays increased deviations between brain regions over time, indicating the implication of miRNAs in the regional specialization as the brain matures (Ziats and Rennert, 2014). Importantly, common neurodevelopmental disorders associated with genes targeted by these miRNAs (Figure 2; Ziats and Rennert, 2014).

Recent elegant work highlights the impact of miRNAs in regulating regionally divergent transcriptional states in the developing human cortex. To characterize the landscape of miRNA–mRNA interactions during human brain development, *Nowakovski et al* developed a new single-cell approach for combined mRNA and miRNA profiling in the same cell across human fetal tissue samples corresponding to peak neurogenesis [Gestational week (GW) 15 and 16.5] and early gliogenesis (GW 19–20.5). Their study revealed that major regulatory molecules like transcription factors, chromatin modifiers, and signaling components are enriched among miRNA targets. Reconstruction of gene-regulatory networks uncovered that miRNA-mRNA interactions often correspond to the acquisition of cell-type identities and undergo dynamic transitions even among closely related cell types during neuronal differentiation and maturation (Figure 2). Further strengthening previous studies, the authors demonstrate that different pathways driven by brain-specific miRNAs are related to developmental stage and cortical area specificity (Nowakowski et al., 2018).

Transcriptomic studies founded the notion that miRNA divergence correlates with the divergence of gene expression patterns in the prefrontal cortex and cerebellum among humans, chimpanzees, and macaques. Accordingly, it has been reported that a significant inverse relationship exists between human and chimpanzee miRNA expression divergence and expression divergence of their predicted target genes at both mRNA and protein levels (Hu et al., 2011). Inline, Svante Pääbo and colleagues placed miRNAs among the key regulators that remodeled cortical development (Somel et al., 2011). Using a computational method of analysis, they show that trans-acting regulators and particularly miRNAs drive the pronounced gene expression changes observed in the human prefrontal cortex. As deduced, the developmental profiles of miRNAs, as well as their target genes, show the fastest rates of human-specific evolutionary change.

## Human- and Primate-Specific Mirnas in Neural Development, Physiology and Pathology

Expression, even if at low levels, has been detected in the prefrontal cortex and cerebellum for the majority of the 14 human-specific miRNAs identified so far (Hu et al., 2012). Enrichment analysis of the predicted target genes of human-specific miRNAs attests that these relate to neuronal components and processes, including cytoskeletal elements, metal ion binding, and postsynaptic, dendritic, and somatic functions (Barbash et al., 2014). Separate studies on individual primate- and human-specific miRNAs are limited and confined in human tissue examination complemented by functional analysis using cell lines or mouse models in one case (Table 2). Nevertheless, data so far show that primate-specific miRNAs are principally involved in the regulation of cell cycle dynamics operating during progenitor proliferation and neuronal differentiation while they further associate with neurodevelopmental disorders (Figure 2).

**Table 2:** A summary of the studies on individual primate- and human-specific miRNAs.

miRNA	Species conservation	mRNA target(s)	Detection of brain expression	Methodology	Proposed associated function and neurodevelopmental disorder	Reference
miR-2115	Primate specific	ORC4	Upregulated at GW19– GW 20 in the human germinal zones	Single-cell RNA sequencing for identification. A mouse model for functional studies	Cell cycle dynamics during human cortical development	Nowakowski et al. (2018)
mir-1301-3p	Primate specific	histone-lysine N-methyl-transferase TCF4 col3a1 and col1a1	Fetal brain of cynomolgus monkeys (Macaca fascicularis, gestation period 165 days)	RNA Sequencing for identification Target validation and luciferase assays in human cell lines	Neurogenesis, schizophrenia, intellectual disability	Arcila et al. (2014)
miR-1180-3p	Primate specific	KANSL1 and DLX1	Fetal brain of cynomolgus monkeys (Macaca fascicularis, gestation period 165 days)	RNA Sequencing for identification Target validation and luciferase assays in human cell lines	GABAergic neurogenesis, autism, intellectual disability	Arcila et al. (2014)
miR-1202	Primate specific	GRM4	Elevated in the brain of depressed individuals	Functional analysis in cell lines and human NPCs. Drug testing in human NPCs.	Pathophysiology of depression	Lopez et al. (2014)
miR-603	Primate specific	E2F1 and LRPAP1		Target validation and luciferase assays in human cell lines	Association with AD risk	Zhang et al. (2016)
miR-941	Human-specific	SMO, SUFU, GLI1, IRS1, PPARGC1A and FOXO1	High expression in the human prefrontal cortex and cerebellum	RNA sequencing for expression analysis. Target validation in human cell lines	Human longevity, neurotransmitter signaling, language and speech	Hu et al. (2012)

A great ape specific miRNA, miR-2115, is enriched in radial glia and becomes prominently upregulated at GW19–20 in the human germinal zones. It controls cell-cycle dynamics during human cortical development by fine-tuning the expression of ORC4, a known regulator of DNA replication (Nowakowski et al., 2018). miR-1301-3p and miR-1180-3p are primate-specific miRNAs that have been identified in the germinal zones of the visual cortex of the macaque developing brain. miR-1301-3p regulates the mRNA for histone-lysine N-methyltransferases mll1 and mll2 (MixedLineage, Leukemia) that function by resolving silenced bivalent loci in neural precursors for induction of neurogenesis. Another target of miR-1301-3p is the transcription factor TCF4 which patterns progenitor cells in the developing CNS and has been linked to schizophrenia and intellectual disability (Arcila et al., 2014). miR-1180-3p targets kansl1 and dlx1. Kansl1 is a chromatin regulator that when haploinsufficient, causes intellectual disability, hypotonia, and distinctive facial features associated with the 17q21.31microdeletion syndrome, while dlx1 is a homeodomain transcription factor that controls GABAergic neurogenesis and has been associated with autism (Arcila et al., 2014).

In addition, a number of species-specific miRNAs associate with human cognition and neurodegenerative diseases (Table 2). miR-941 is the only human-specific miRNA expressed highly in the prefrontal cortex and cerebellum and has been proposed to be associated with longevity and neurotransmitter signaling. Individuals containing a microdeletion in the chromosomal region containing pre-miR-941 display developmental delay and disruption of cognitive functions including language and speech (Figure 2; Hu et al., 2012). miR-1202, a miRNA specific to primates, and enriched in the human brain is associated with the pathophysiology of depression. It is differentially expressed in depressed individuals and has been shown to target the Metabotropic Glutamate Receptor 4 (GRM4; Lindsley and Hopkins, 2012), a synaptic molecule modulating neurotransmission that also constitutes an attractive therapeutic target for Parkinson’s disease and schizophrenia (Figure 2; Lopez et al., 2014).

Another primate-specific miRNA, miR-603, is a novel intronic miRNA of the gene KIAA1217, which is highly expressed in human brain. miR-603 directly downregulates the key neuronal apoptotic component E2F1 and can prevent cells from undergoing apoptosis. In addition, miR-603 targets LRPAP1 involved in Aβ amyloid peptide clearance and the pathogenesis of AD. Finally, the rs11014002 SNP in precursor pre-miR-603 increases the expression of mature miR-603, which may account for its association with reduced risk for AD (Zhang et al., 2016; Figure 2).

## Human-Specific Regulation of Mirna Function in the Brain

Evolutionary changes and consequently species distinct features also exist for the binding partners of miRNAs. The evolution of miRNA regulation is intimately intertwined with the evolution of their targets, as newly emerging miRNAs integrate into preexisting gene expression circuitries (Arcila et al., 2014). This is particularly pronounced in the brain where neuronal transcripts not only have longer 3′-untranslated regions (UTRs) which are the main target region for miRNAs (Meunier et al., 2013), but also display an increased density of potential binding sites, enhancing their selective advantage for acquiring miRNA-mediated regulation (Cacchiarelli et al., 2008; Barbash et al., 2014). These findings in conjunction with the appearance of increased new variants around target genes of human-specific miRNAs are consistent with the theory that the speciation of hominids was accompanied by an enhancement in the capacity of newly evolved miRNAs to modulate gene expression (Barbash et al., 2014).

Interestingly Hu et al identified five miRNAs, namely miR-184, miR-487a, miR-383, miR-34c-5p, and miR-299-3p, with high sequence conservation among species, which nevertheless show significantly different levels of expression in humans, while two of these (miR-299-3p and miR-184) show preferential expression in cortical neurons (Hu et al., 2012). Functional analysis combining the targets of all five miRNAs revealed enrichment in genes associated with neural processes and specifically with cell proliferation and differentiation, synaptic transmission and neuronal function. Moreover, miR-299-3p targets associate with axon guidance, while miR-184 targets are related to long-term potentiation, which is directly linked to learning and memory formation (Hu et al., 2011). Human-specific miRNA-mediated regulation of certain genes with a critical role in brain function has also been reported. For instance, miR-483–5p, binds in a sequence-exclusive manner to the human epigenetic regulator methyl CpG binding protein 2 (MeCP2) that controls proper neurological function, to modulate its levels in the fetal cortex (Han et al., 2013).

## Human Experimental Models for Investigation of Mirna-Specific Features of Brain Development and Associated Disorders

Access to human tissue in combination with high-throughput sequencing techniques provided important insight into the significance of the miRNA-driven transcriptome changes across brain development and evolution. However, it is necessary to validate predicted targets and clarify cell type association, stage-specificity and mode of action for individual miRNAs. As experimental animals cannot fully simulate human brain development and disease, human embryonic stem cells (hESCs) or induced pluripotent stem cells (iPSCs) represent valuable means for advancing human studies (Chambers et al., 2009; Shi et al., 2012). Directed cortical differentiation of human, chimpanzee, and macaque iPSCs (Otani et al., 2016) clearly depicted the divergence in the timing of key developmental events and highlighted the extended proliferation of chimpanzee and human progenitors as compared to macaque, emphasizing the validity of such systems in reproducing species-specific features.

Further studies on hESCs accompanied by miRNA profiling during differentiation of various neuronal subtypes underlined the effect of known miRNAs, such as let-7, miR-124, miR-7, miR-125 and miR-9 in progenitor proliferation, cell fate specification and neuronal commitment and maturation (Delaloy et al., 2010; Boissart et al., 2012; Liu et al., 2012; Cimadamore et al., 2013; Tu et al., 2018). miRNA research on hESC models has also uncovered new targets and roles for these miRNAs. For example, miR-9 which has been described in mouse to promote neuronal lineage differentiation by targeting REST, was shown to also target Stathmin, a protein involved in microtubule stability, and to coordinate proliferation and migration during the early stage of maturation of neural progenitors. Similarly, miR-125 with reported neuronal function, was shown to act in earlier stages to promote exit from pluripotency and potentiate neural specification by targeting SMAD4. Moreover, research on patient induced pluripotent stem cells and their neuronal derivatives uncovered the role of certain miRNAs in autism and schizophrenia (Halevy et al., 2015; Murai et al., 2016; Mellios et al., 2018).

Notably, given the complexity of the human brain, 3D tissue culture models that embody cellular diversity and spatial organization that mimics brain architecture, are more relevant to understand the critical input of miRNAs in shaping intricate brain gene networks. Brain organoids that grow as 3D aggregates from pluripotent stem cells comprise an exciting new tool for modeling human brain physiology and pathology, for uncovering human-specific traits and for drug discovery and testing. Despite current limitations, particularly batch heterogeneity that impedes consistency, organoids resemble human brain not only at the cellular level, but also in terms of general tissue structure, developmental trajectories and neuronal functionalities (Lancaster et al., 2013; Paşca et al., 2015; Qian et al., 2016; Giandomenico et al., 2019). Cortical organoids in particular that have been more extensively studied, recapitulate the organization of neural progenitor zones to a considerable degree, reflecting developmental events during embryonic stages *in vivo* (Lancaster et al., 2013; Qian et al., 2016). Such systems can be valuable for functional gain- or loss-of-function studies to elucidate the contribution of ancient or novel miRNAs in primate and/or human-specific processes. Nevertheless, studying neuronal network formation among different brain regions, especially distant ones, remains a challenge. Recent advances in the generation of fused brain organoids from co-culture of individual ones with distinct regional identities may provide a *closer-to-the-in-vivo-situation* model to investigate the basis of human neural circuitry formation in health and disease (Birey et al., 2017; Xiang et al., 2017, 2019). Alternatively, it is possible to exploit the inherent intrinsic heterogeneity observed within single organoids, as for example in brain organoids containing retinal regions that can respond to light stimuli (Quadrato et al., 2017). However in all cases absence of vascularization restricts oxygen and nutrient supplies limiting organoid survival, consequently affecting the time-scale of such studies. Therefore a combination of different approaches and technologies, including 2D cultures derived from pluripotent stem cells, brain organoids, single-cell transcriptomics, potentially along with miRNA target degradation kinetics analysis to elucidate the miRNA-driven evolutionary variants of the neurodevelopmental program should prove more rewarding.

In relevance, single-cell RNA-sequencing technology has greatly facilitated research efforts in resolving the diversity and developmental trajectories of human brain cell types providing transformative insights into the developmental lineages and functional states of individual cells (Tasic et al., 2016, 2018; Nowakowski et al., 2017; Paul et al., 2017; Hrvatin et al., 2018; Lake et al., 2018; Zhong et al., 2018). Importantly, a number of studies have illustrated the potential to integrate spatial information at single-cell resolution (Ke et al., 2013; Lubeck et al., 2014; Chen et al., 2015; Satija et al., 2015; Salmén et al., 2018). However, when designing such experiments, consideration of the tradeoff between a number of cells sequenced and the read depth per cell is a factor that has to be seriously considered, not the least because of budgetary constraints (Menon, 2018). Additionally, such developments still necessitate the formulation of new computational modalities to integrate the rapidly expanding data sets generated from diverse sources so that biological sense is made (Butler et al., 2018; Stuart and Satija, 2019). Improvements in algorithm design and data analysis are essential to allow unsupervised methods for tracking gene expression and correctly reconstructing trajectories that map cell lineages and developmental transition of cells.

### Towards miRNA Based Therapeutics

The more we understand miRNA biology and its contribution to the intricate regulatory networks of gene expression during development, the better will we be able to uncover related disturbances underlying the origin of various neuropsychiatric and behavioral disorders. Indeed, compelling evidence regarding the involvement of miRNAs in human disease has instigated attention into their potential use as therapeutics (van Rooij et al., 2012). miRNA-based strategies include miRNA mimics and inhibitors (antagomiRs) to respectively increase and decrease their expression or, conversely, their target genes. The field currently progresses rapidly as in 2018 the FDA approved the first therapy based on miRNA administration for the treatment of rare progressive polyneuropathy caused by hereditary transthyretin-mediated amyloidosis (Adams et al., 2018; Wood, 2018). Understanding the functions of miRNAs in specific cell types and during different stages throughout life under normal and pathological conditions could improve the specificity and efficacy of miRNA therapeutic strategies. Moreover, delivery to the brain is challenging and the pleiotropic nature of miRNA functions undeniably makes off-target biological effects an important limitation (Junn and Mouradian, 2012). Novel approaches are in the pipeline to design new chemical formulations in order to increase miRNA stability and improve their specificity and permeability, while delivery methods are also being developed to target the brain and decrease unwanted side effects (van Rooij et al., 2012; Søkilde et al., 2015; Rupaimoole and Slack, 2017). In this regard, decoding species-specific regulation of miRNA function will prove an important step for fostering novel, holistic and effective therapeutic approaches for human disorders.

## Conclusions and Future Perspectives

Accumulating evidence highlights the emergent role of miRNAs as critical regulators that influence the overall transcriptional landscape in the human brain. In addition, primate- and human-specific regulation by miRNAs during neurodevelopment argues in favor of their involvement in the establishment of species-specific features during brain evolution. A comprehensive understanding of the spatiotemporal miRNA mediated regulation will help resolve the intricate regulatory network operating during human neurogenesis and will grant important cues for the causality of neurodevelopmental disorders. Down this line, 2D- and 3D-human pluripotent-derived experimental setups constitute a useful system for modeling species-specific features of development and for capturing miRNA mediated rewiring of the transcriptional program. In conjunction, single-cell sequencing approaches can decode the refined miRNA-mRNA interactions during cell developmental transitions. Taken together, miRNAs constitute an indispensable component of the extended gene regulatory network and evaluating their functional significance in the evolving brain can determine key features of human neuronal development and disease.

## Author Contributions

KP and RM planned and wrote the manuscript.

## Conflict of Interest

The authors declare that the research was conducted in the absence of any commercial or financial relationships that could be construed as a potential conflict of interest.
